# Autoimmune glial fibrillary acidic protein astrocytopathy: clinical analysis and review of 15 cases

**DOI:** 10.1007/s13760-023-02268-0

**Published:** 2023-04-20

**Authors:** Wei Lan, Jiming Li, Peiying Ai, Weiliang Luo

**Affiliations:** Department of Neurology, Huizhou Central People’s Hospital, No. 41, Eling North Road, Huizhou, 516001 Guangdong China

**Keywords:** Anti-glial fibrillary acidic protein antibody, Antibody titer, Dual clinical symptoms, Treatment effect

## Abstract

**Background:**

To review clinical characteristics, auxiliary examination results, treatment effects, and outcomes of patients with autoimmune glial fibrillary acidic protein astrocytopathy (GFAP-A).

**Methods:**

We collated and retrospectively analyzed clinical data of 15 patients admitted with clinical characteristics of an autoimmune GFAP-A acute encephalitis or meningitis phenotype.

**Results:**

All patients were diagnosed with acute-onset meningoencephalitis and meningoencephalomyelitis. Initial presentations included pyrexia and headache at onset; dual symptoms of prominent tremor with urinary and bowel dysfunction; ataxia, psychiatric and behavioral abnormalities, and impaired consciousness; neck resistance; reduced extremity muscle strength; blurred vision; epileptic seizures; and reduced basic blood pressure. Cerebrospinal fluid (CSF) examination showed that the degree of protein elevation was significantly higher than the degree of increase in white blood cells. Moreover, in the absence of obvious low chloride and glucose levels, CSF chloride levels decreased in 13 patients, accompanied by a CSF glucose level decrease in four. Brain abnormalities were found in magnetic resonance imaging of ten patients, with a linear radial perivascular enhancement present in the lateral ventricles of two patients and symmetric abnormalities in the splenium of the corpus callosum in three patients.

**Conclusions:**

Autoimmune GFAP-A may be a spectrum disorder, with acute- or subacute-onset meningitis, encephalitis, and myelitis being the main phenotypes. When used for acute stage treatment, combined hormone and immunoglobulin therapy was superior to hormone pulse therapy or immunoglobulin pulse therapy alone. However, hormone pulse therapy alone without immunoglobulin pulse therapy was associated with a greater number of remaining neurological deficits.

## Introduction

In recent years, autoimmune encephalitis or encephalopathy has attracted considerable interest from researchers around the world. Autoimmune glial fibrillary acidic protein (GFAP) astrocytopathy (GFAP-A) is an inflammatory autoimmune disorder of the central nervous system that was first described in humans in 2016. Currently, GFAP immunoglobulin G (GFAP-IgG) is regarded as a specific biomarker for the diagnosis of autoimmune GFAP-A [1, 2]. However, it is debated whether anti-GFAP antibodies possess pathogenicity. Although GFAP-associated encephalitis has been reported in the literature, evidence related to the clinical course, recurrence, and outcomes of the disease remains lacking.

To address this issue, we performed a retrospective analysis of the clinical data of 15 patients admitted to our hospital who were anti-GFAP antibody-positive and manifested acute encephalitis or meningitis, with the aim of determining the clinical characteristics and outcomes of such patients to provide a scientific basis for future clinical diagnoses.

## Methods

### Study participants

Ethics approval for this study was obtained from Huizhou Central People’s Hospital. Twenty-three patients admitted to Huizhou Central People’s Hospital between March 1, 2020, and January 31, 2022, whose serum and cerebrospinal fluid (CSF) samples were screened. The inclusion criteria were patients with acute disease onset, with clinical presentations consistent with encephalitis, meningitis, myelitis, or a combination of the above; and CSF anti-GFAP-IgG antibody positivity. The exclusion criterion was a definite diagnosis of other diseases. Neoplastic diseases were ruled out by completing tumor marker screening, such as AFP, CEA, PSA, CA125, CA199, CA153, etc., and undertaking lung and abdominal imaging (CT or Ultrasound). Among the 23 patients, eight patients with anti-GFAP antibody positivity in serum and an absence of the aforementioned symptoms were excluded from the study due to the following: diagnosis of brain abscess (*n* = 1), positivity for antibodies in nodes of Ranvier and multiple antibodies against gangliosides (*n* = 1), diagnosis of neuromyelitis optica (*n* = 3), bilateral medullary infarction (*n* = 1), central pontine myelinolysis (*n* = 1), and positivity for multiple paraneoplastic antibodies (*n* = 1). Fifteen patients were ultimately included in the present study.

### Data collection

GFAP-IgG was tested by a cell-based assay (CBA). HEK293 cells were co-transfected with full-length human GFAP and pcDNA3.1-EGFP according to a previous report [3].

All cases were improved with at least one autoimmune encephalitis-related antibody, paraneoplastic syndrome-related antibodies, and metagenomic next-generation sequencing screen. Including anti-glutamate receptor (NMDA type) antibody, Anti-glutamate receptor (AMPA type 1) antibody, Anti-glutamate receptor (AMPA type 2) antibody, anti-leucine-rich glioma inactivating protein 1 (LGI 1) antibody, Anti-contact protein-associated protein-2 (CASPR2) antibody, Anti-γ-aminobutyric acid type B receptor (GABAB) antibody, anti-IgLON family protein 5 (IgLON5) antibody anti-dystrophin-like protein 6 (DPPX) antibody, anti-glycine receptor 1 (GlyR1) antibody, anti-dopamine receptor 2 (DRD2) antibody, anti-glutamate decarboxylase 2 (GAD65) antibody, anti-metabotropic glutamate receptor 5 (mGluR5) Antibody, Anti-metabotropic glutamate receptor 1 (mGluR1) Antibody, Anti-synuclein-3α (Neurexin-3α) Antibody, Anti-gamma-aminobutyric acid type A receptor (GABAA) Antibody, Anti-Kelch-like protein 11 (KLHL11) Antibody, anti-myelin oligodendrocyte glycoprotein (MOG), anti-myelin basic protein (MBP),anti-Hu, anti-Ri, anti-CV2, anti-Amphiphysin, anti-Ma1, anti-Ma2, anti-SOX1, anti-Tr (DNER), anti Zic4 antibody, Titin antibody, Recoverin antibody, anti-PKCγ antibody, anti-Yo antibody, and anti-glutamic acid decarboxylase 2 (GAD65) antibody. Detection was carried out as described in the previous studies [4, 5].

The following data were collected for the 15 patients for retrospective analysis: demographic characteristics, clinical presentations (initial symptoms, main symptoms, and accompanying symptoms), auxiliary examinations (routine blood test, biochemical test, thyroid function, tumor markers, etiological indicators, CSF examination, electroencephalography [EEG], electromyography [EMG], and head magnetic resonance imaging [MRI]), treatment regimens, and outcomes.

### Follow-up and evaluation methods

Patient outcomes were determined through a standardized questionnaire and during periodic re-hospitalization and outpatient follow-up. The modified Rankin scale (mRS) was used to evaluate the severity of symptoms and functional outcomes of the patients during the fastigium and at the 1-month, 2-month, and final follow-ups. mRS scores ranged from 0 to 6, with each score indicating the following: 0, no symptoms at all; 1, no significant disability, able to carry out all usual activities despite some symptoms; 2, slight disability, able to look after own affairs without assistance, but unable to carry out all previous activities; 3, moderate disability, requires some help but able to walk unassisted; 4, moderately severe disability, unable to attend to own bodily needs without assistance, or unable to walk unassisted; 5, severe disability, requires constant nursing care and attention, bedridden, incontinent; 6, deceased [6].

## Results

### Clinical characteristics

Table 1 shows the clinical characteristics of the 15 autoimmune GFAP-A patients. Disease onset occurred across a wide range of ages, with the oldest and youngest patients aged 85 years and 14 years, respectively, and the mean age being 47.3 years. The majority were male (male:female ratio = 2:1). Clinical presentations were varied, with acute- or subacute-onset meningitis, encephalitis, myelitis, or meningoencephalomyelitis being the main presentations and with isolated myelitis being relatively rare. All 15 patients experienced pyrexia, headache, extremity tremor, urinary and bowel dysfunction, neck resistance, and blurred vision. Other common manifestations included ataxia (13/15), psychiatric abnormalities (9/15), convulsions (7/15), and impaired consciousness (10/15).

**Table 1:** Clinical characteristics of the 15 patients

Patient no	Age (years)	Sex	Chief complaint at admission	Duration of pyrexia (days)	Clinical characteristics												Other accompanying symptoms and special comorbidities
					Pyrexia	Headache	Tremor	Urinary dysfunction	Bowel dysfunction	Ataxia	Neck resistance	Psychiatric abnormalities	Impaired consciousness	Convulsions	Extremity weakness	Blurred vision	
1	14	Male	Pyrexia and headache for 9 days, extremity tremor for 1 day	64	+	+	+	+	+	+	+	+	+	+	+	+	Refractory hypotension, numbness in the ends of extremities, mechanical ventilation
2	51	Male	Pyrexia and headache for 1 month, nonsensical speech for 20 days	32	+	+	+	+	+	+	+	+	+	+	+	+	Numbness in the ends of extremities, mechanical ventilation
3	52	Male	Pyrexia for 10 days	19	+	+	+	+	+	+	+	–	–	–	+	+	Numbness in the ends of extremities
4	54	Female	Pyrexia, headache for 6 days	12	+	+	+	+	+	–	–	–	–	–	+	+	Nil
5	86	Male	Headache for 1 day, weakness in the right lower extremity for 12 h	53	+	+	+	+	+	+	+	+	+	+	+	+	Hypotension, mechanical ventilation
6	49	Male	Intermittent pyrexia for 2 months	87	+	+	+	+	+	+	+	–	+	–	+	+	Cough, tinnitus
7	65	Male	Headache and pyrexia accompanied by weakness in the left extremities for 5 days	5	+	+	+	+	+	–	–	–	–	–	+	+	Nil
8	27	Male	Recurrent pyrexia for more than 10 days, abdominal pain and vomiting for more than 1 week	12	+	+	+	+	+	+	+	+	+	–	+	+	Incomplete intestinal obstruction, peritonitis, acute renal failure, ascites
9	58	Male	Dizziness and headache for 1 week	-	–	+	–	–	–	+	–	–	+	–	+	+	Nil
10	48	Female	Recurrent headache for 5 days, convulsive seizures for 2 days	6	+	+	+	+	+	+	√	√	+	+	+	+	Nil
11	14	Female	Pyrexia and headache for 10 days	17	+	+	+	+	+	+	+	+	+	+	+	+	Incomplete intestinal obstruction
12	47	Female	Pyrexia and headache for 20 days, altered state of consciousness for 1 day	30	+	+	+	+	+	+	+	+	+	+	+	+	Nil
13	50	Male	Pyrexia and headache for more than 1 week	7	+	+	+	+	+	+	+	+	+	–	+	+	Numbness in the ends of extremities
14	Male	Male	Pyrexia and headache for 9 days, psychiatric abnormalities for 1 day	27	+	+	+	+	+	+	+	+	+	–	+	+	Mechanical ventilation, pneumothorax
15	30	Male	Headache for 8 days, pyrexia for 6 days	15 + ?	+	+	+	+	+	+	+	+	+	–	+	+	Mechanical ventilation, incomplete intestinal obstruction

### Laboratory examinations

#### CSF examination

CSF pressure showed a mild-to-moderate increase (> 180 mmH_2_O) in the majority of patients (10/15; Table 2). The elevation in CSF albumin levels was significant and exceeded 1.0 g/L in most patients (12/15). However, the increase in white blood cell (WBC) count was less than 300 × 10^6^/L (10/15). Cytological examination of the CSF, which was performed in four patients, revealed the predominance of lymphocytes. A reduction in the chloride level of CSF was common (13/15), with three patients showing concurrent decreases in glucose and chloride levels.

**Table 2:** Clinical characteristics of the 15 patients determined from the first lumbar puncture

Patient No	Age (years)	Sex	Pressure (mmH_2_O)	Color	Albumin (mg/L)	Cell count (× 10^6^/L)	Glucose (mmol/L)	Chlorides (mmol/L)
1	14	Male	280	Pale yellow	1430.8	60	3	113.3
2	51	Male	270	Pale yellow	2680	80	1.77	117
3	52	Male	130	Clear	1502	137	2.62	119.6
4	54	Female	160	Clear	1155	298	3.88	115.9
5	86	Male	190	Clear	2496	72	4.18	114.3
6	49	Male	186	Clear	1520	240	2.4	112.8
7	65	Male	158	Clear	693	3	3.55	117.8
8	27	Male	100	Clear	1159	30	2.84	117
9	58	Male	188	Clear	595	13	6.26	120.5
10	48	Female	182	Clear	242	1	4.13	124.2
11	14	Female	190	Clear	1182	293	2.2	117.9
12	47	Female	310	Clear	1360	278	1.26	117.5
13	50	Male	110	Pale yellow and transparent	2169	64	2.88	114.4
14	Male	Male	205	Pale yellow and transparent	2585	142	2.16	114.3
15	30	Male	270	Clear	775	108	3.6	113.7

#### Anti-GFAP antibody

All 15 patients exhibited anti-GFAP antibody positivity in the serum and CSF. Two patients tested negative in the serum and CSF during the first lumbar puncture, performed within 7 days of admission. The CSF results of two patients were negative during the initial examination and positive upon re-examination. Follow-up antibody re-examination results of four patients revealed that the serum antibody titer turned negative before that of CSF. The longest time to negative conversion in CSF and serum was 134 days and 75 days, respectively (Table 3).

**Table 3:** Characteristics of GFAP antibody titer change

Patient No	Time of first positive CSF result—time of disease onset (days)	Time of first positive result in serum (days)	First antibody titer in CSF	First antibody titer in serum	Time to negative conversion in CSF (days)	Time to negative conversion in serum (days)	OB test result
1	12	12	1:100	1:32	134	46	Neg
2	17	17	1:10	Neg	–	–	Neg
3	21	21	1:32	1:32	–	–	Neg
4	10	10	1:32	1:32	–	20	Neg
5^a^	25	25	1:100	1:32	–	38	Neg
6	36	36	1:1000	–	75	75	Neg
7	5	5	1:32	1:100	15	15	Neg
8	17	17	1:100	1:32	–	–	Neg
9	12	12	1:32	1:32	–	–	Neg
10^b^	5	5	Neg	1:100	–	–	Neg
11	14	–	1:32	Neg	–	–	Neg
12	28	–	1:32	–	–	–	Neg
13	7	7	1:32	1:32	–	–	Neg
14^a^	19	19	Neg	Neg	–	–	Pos
15	11	–	1:1	Neg	–	–	Neg

*CSF* cerebrospinal fluid, *OB* oligoclonal bands

^a^Normal GFAP results during first CSF and serum examination

^b^Negative result in CSF during first examination and positive result in CSF during re-examination

#### Etiological testing

All patients underwent metagenomic next-generation sequencing (mNGS) of CSF at least once for the identification of microbes. Epstein–Barr virus (EBV) positivity was found in seven patients (7/15), but only a small number of sequences were detected in all cases (maximum 8 copies). Standard EB-DNA testing at our hospital by polymerase chain reaction (PCR) also indicated EBV-positivity in these seven patients. Lyme disease antibody positivity and concomitant *Streptococcus intermedius* infection (number of mNGS sequences: 4) were found in one patient each. The T-SPOT.TB test was performed on the blood samples of ten patients, and results indicated positivity in two patients. All patients were subjected to CSF culture, smear, herpes simplex virus-DNA, tuberculosis (TB)-DNA, cytomegalovirus-DNA, and cryptococcus testing, but no abnormalities were shown. Other serological tests, such as the Weil–Felix test, Widal test, mycoplasma test, chlamydia test, respiratory pathogen panel (nine pathogens), and fungal antigen test also revealed no abnormalities.

#### Inflammatory indicators

Serum WBC counts of the patients during the first test were almost normal, with elevated WBC counts were found in two patients with concomitant lung inflammation. Some patients exhibited a mild increase in monocytes, particularly those with concomitant EBV infection, while others showed a mild increase in eosinophil levels. Hypersensitive C-reactive protein was completely normal during the initial disease onset and 1-week follow-up (13/15), with mild increases occurring in two patients with concomitant mild lung inflammation. Similarly, procalcitonin was almost normal or showed a borderline mild increase of not more than three times the upper limit of normal. One patient may have had concomitant respiratory infection, while the other 12 patients had no obvious infection foci. The erythrocyte sedimentation rate was normal in almost all patients (14/15), with a mild increase observed in one patient who may have had concomitant immune abnormalities induced by other antibodies (Tables 4, 5).

**Table 4:** Characteristics of inflammatory markers

Patient No	First serum WBC count (× 10^9^/L)	Second serum WBC count (× 10^9^/L)	First CRP (2.5 mg/L)^a^	Second CRP (2.5 mg/L)^a^	First PCT (ng/L)^b^	Second PCT (ng/L)^b^	ESR (mm·h)^c^	Other concomitant non-CNS infections
1	5.6	9.9	N	N	N	0.07	N	Nil
2	8.3	9.8	N	N	N	(–)	N	Nil
3	7.8	6.6	N	N	0.082	N	N	Lyme disease
4	4.82	4	N	N	0.11	(–)	N	Nil
5	12.1	16.5	N	8.02	(–)	0.139	N	Mild lung inflammation
6	8.11	(–)	N	(–)	N	(–)	(–)	Nil
7	6.9	6.4	131.63	17.65	0.12	0.05	32	Anti-contactin-associated protein-2 (CASPR2) IgG antibodies: ( +) 1:10
8	10.1	8.4	27.69	1.33	1.33	(–)	N	Mild lung inflammation
9	10.9	10.1	N	(–)	0.062	(–)	N	Nil
10	17.33	14	N	N	0.056	0.05	N	Mild lung inflammation
11	7.33	6.7	N	N	N	(–)	N	Nil
12	4.7	3.7	N	-	0.6	(–)	N	mNGS increased TB
13	6.07	5.18	N	N	64	0.06	(–)	Nil
14	7.9	5.53	N	N	0.07	N	(–)	Nil
15	6.67	10.07	N	N	0.11	0.008	3.6	Nil

*N* reference test values are within the normal range, (–) missing data

*CNS* central nervous system, *WBC* white blood cell, *CRP* C-reactive protein, *PCT* procalcitonin, *ESR* erythrocyte sedimentation rate

^a^Normal reference value < 2.5 mg/L

^b^Normal reference value < 0.046 ng/L

^c^Normal reference value < 10 mm·h

**Table 5:** MRI and EEG characteristics of 15 patients with autoimmune GFAP astrocytosis

Patient No	Magnetic resonance imaging (MRI)	Electroencephalography (EEG)
1	Widespread symmetric abnormalities in the corpus callosum, bilateral posterior limbs of the internal capsule and thalamus, and cerebral peduncle—medulla oblongata, suspicious slight swelling in the cervical spinal cord, significant enhancement in the cervical spinal cord and spinal meninges of multiple segments, no abnormalities observed in the NAA, Cho, and Cr peak forms, no significant increases or decreases, absence of abnormal wave peaks	Generalized moderately abnormal EEG (diffuse slowing of background activity, persistent generalized diffuse occurrences of 1.0–3.0 Hz irregular slow waves and slow activity in various leads, mainly in the frontal region)
2	Mild linear radial perivascular enhancement pattern in the lateral ventricles	Normal during the early stage, mildly abnormal EEG, increased θ waves observed in various leads
3	Normal	Normal
4	Normal	Normal
5	Only head computed tomography was performed, MRI was not performed	Generalized moderately abnormal EEG
6	Hydrocephalus in the bilateral lateral and third ventricles accompanied by mild interstitial brain edema around the lateral ventricles, collapsed fourth ventricle, obstructive hydrocephalus (site of obstruction: central aqueduct) was considered, possibly caused by local ependymal adhesion. Diffuse enhancement in intracranial brain surface meninges and cervical spinal meninges, dilatation of deep medullary veins, cerebral and spinal meningitis were considered	Generalized moderately abnormal EEG
7	Normal	Normal
8	Normal	Borderline EEG
9	1. Re-examination of brain abscess: significant shrinkage of enhanced lesions in the right parietal and occipital regions and the surrounding edema and a smaller space-occupying effect compared to the previous examination; 2. Same brain stem signal abnormalities as before, the possibility of ischemic foci or demyelinating lesions should be considered	Moderately abnormal EEG, lack of symmetry in background activity, with a significant reduction in the frequency and amplitude of the α rhythm in the right occipital region compared with the left occipital region. Sporadic or episodic 2.0–3.0 Hz irregular slow wave discharges were frequently observed in the right frontal pole and frontal, anterior temporal and middle temporal regions
10	Normal	Moderately abnormal EEG: small number of sporadic discharges of irregular slow waves in the right frontal pole and anterior temporal region, abnormal EEG evolution mainly occurring in the right frontal pole and frontal region were detected twice (attention should be paid to non-clinical seizures)
11	Slight thickening of bilateral pia mater, small patch-like abnormal signals in the splenium of the corpus callosum, attention should be paid to meningitis accompanied by reversible lesions in the splenium of the corpus callosum	Abnormal EEG: increased slow background activity, 2–3-Hz slow wave discharges in the right frontal pole, frontal and temporal regions or left parietal, occipital, and temporal regions
12	Multiple abnormal-signal nodules in the right occipital lobe, corona radiata, and cerebellar hemisphere, the nature of which is to be determined (metastatic neoplasms/ inflammatory nodules/others, to be correlated with clinical findings)	Nil
13	Normal	Nil
14	Signal abnormalities in the splenium of the corpus callosum	Nil
15	Signal abnormalities in the splenium of the corpus callosum and thoracic spinal cord	Nil

#### Other antibody tests

Two patients tested positive for autoimmune encephalitis antibodies, with one patient having concomitant anti-CASPR2 antibody positivity in both the serum and CSF (titer of 1:10 in both serum and CSF) and the other patient having anti-LGI1 antibody positivity in both the serum (titer of 1:30) and CSF (titer of 1:10). One patient exhibited anti-sulfatide antibody positivity in serum. No abnormalities were observed in the test results for anti-APQ4, anti-MOG, anti-MBP, and paraneoplastic antibodies and the purified protein-derivative skin test, performed in 13 patients. Oligoclonal band-positivity was found in two patients. Test results for five types of antineutrophil cytoplasmic antibodies and the comprehensive autoimmune antibody panel (antinuclear antibodies, anti-double stranded DNA [dsDNA] antibodies, extractable nuclear antigen antibodies, anti-Smith [Sm] antibodies, anti-ribonucleoprotein [RNP] antibodies, anti-SSA antibodies, anti-SSB antibodies, and anti-Scl 70 antibodies) were mostly normal (10/15), with two patients testing weakly positive for antibodies to nRNP/Sm and two patients testing weakly positive for antibodies to SAA and RO-52. In the thyroid antibody test (anti-thyroid peroxidase [TPOAb], anti-thyroglobulin [TgAb], and anti-thyrotropin receptor antibodies), one patient exhibited significant elevations in TPOAb and TgAb.

### Electrophysiological examination

Non-specific changes were observed in the EEGs of the patients. These mainly manifested as diffuse slow waves. Eight patients had abnormal EEGs, of which seven exhibited generalized moderately abnormal EEG changes without obvious epileptiform discharges. Results of evoked potential testing performed in ten patients revealed the presence of bilateral visually evoked potentials and auditory brainstem response abnormalities in some patients (3/10 and 2/10, respectively).

### Imaging characteristics

**Fig. 1:**
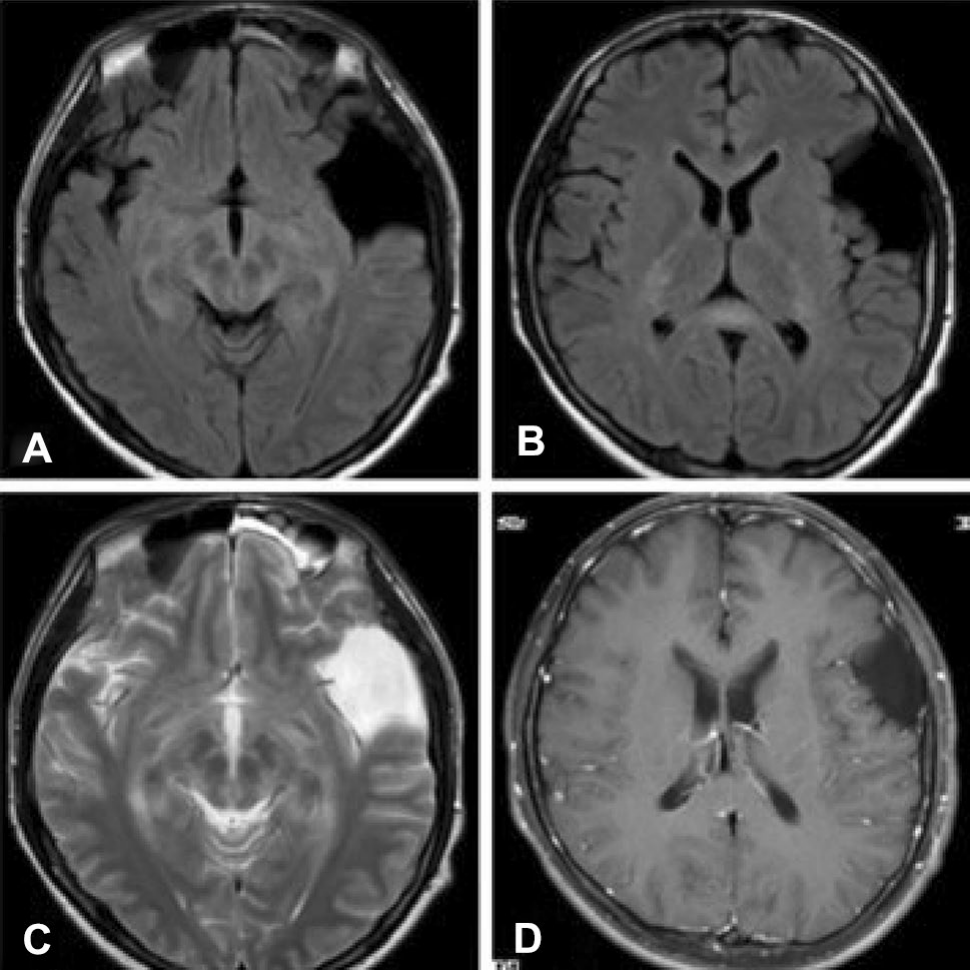
1 MRI of case 1: FLAIR visible hippocampal, thalamic, and midbrain high signal (**A**); callosal compression lesion (**B**); T2 sequence visible hippocampal, thalamic, and midbrain high signal (**C**); T1 enhancement visible as a discharge-like strengthening sign a discharge-like strengthening sign around the lateral ventricles, with no signal strengthening in the corpus callosum (**D**) (note: the patient had a similar lamellar high signal in the cervical medulla T2)

**Fig. 2:**
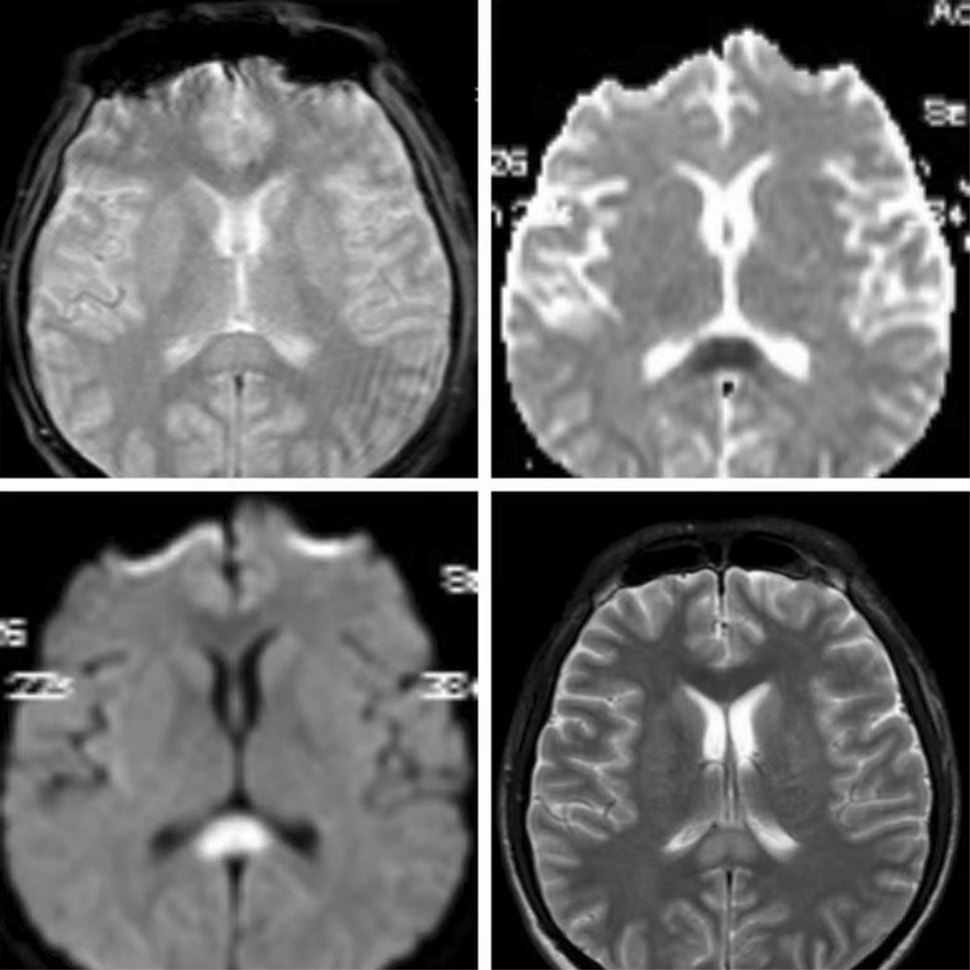
MRI of case 14: abnormal signal of the corpus callosum visible (note: two other patients had similar signal abnormalities in the corpus callosum)

Six patients had normal imaging characteristics (6/15) and three patients had MRI abnormalities. Two patients exhibited a mild linear radial perivascular enhancement pattern in the lateral ventricles (2/15). Three patients had concomitant symmetric abnormalities in the splenium of the corpus callosum (Figs. 1, 2), two patients had concomitant spinal cord lesions, one patient had secondary hydrocephalus induced by a long disease course, one patient had concomitant brain abscess, and one patient had concomitant vestibular schwannoma. Positron-emission tomography scanning performed on 3 of the 15 patients revealed an absence of tumor manifestations.

### Treatment and follow-up

The longest follow-up duration for the patients in the present study was 2 years, and recurrence has not been observed to date. Patient 1 experienced residual mild detrusor dysfunction and paresthesia below the thoracic plane. Patient 5 had a worse state of consciousness and poorer muscle strength recovery in the extremities due to old age, which necessitated tracheotomy, but not ventilator assistance. Subsequently, he developed concomitant fungal infection and was prescribed chronic bed rest. Patient 13 was administered antiviral therapy with acyclovir, and normal-dose dexamethasone was also used due to patient refusal of immunoglobulin therapy. After a disease course of 3 months, the patient still experienced residual voiding dysfunction and physical weakness (mainly in the lower extremities) and required long‐term urinary catheterization. Patients 1, 2, 14, and 15 experienced rapid progression and developed quadriplegia and type 2 respiratory failure within a short period of time, which necessitated intubation and ventilator support. However, the patients recovered rapidly after immunoglobulin pulse therapy and had satisfactory outcomes (Table 6).

**Table 6:** Treatment and follow-up

Patient no	Initial regimen	Modified regimen	Pathogen	Concomitant anti-neuronal antibody	Follow-up (mRS)			Outcome
					1 month	2 months	6 months	
1	Acyclovir + antibiotic	Immunoglobulin + methylprednisolone hormone pulse therapy	EBV	Nil	3	2	1	Satisfactory
2	Acyclovir + antibiotic + antitubercular agent + methylprednisolone sodium succinate pulse therapy + immunoglobulin	Antitubercular agent were stopped, hormone dose was reduced	Nil	Nil	1	1	0	Good
3	Antibiotic	Antibiotic + immunoglobulin	*Borrelia burgdorferi*	Nil	1	0	0	Good
4	Antibiotic + acyclovir	Immunoglobulin	Nil	Nil	0	0	0	Cured
5	Antiviral + antibiotic	Immunoglobulin + hormone	EBV	Nil	5	5	5	Poor
6	Antiviral + antibiotic + antitubercular agent + immunoglobulin	Hormone pulse therapy	Nil	Nil	2	1	1	Good
7	Antiviral + antibiotic	Nil	EBV	CASPR2 antibody	1	0	0	Good
8	Antibiotic	Nil	EBV	Nil	0	0	0	Good
9	Antibiotic	Nil	*Streptococcus intermedius*		0	0	0	Good
10	Antibiotic + antiviral	Nil	Nil	LGI1 antibody	1	0	0	Good
11	Antibiotic + antiviral + immunoglobulin + hormone	Nil	EBV	Nil	1	0	0	Good
12	Antibiotic + Antiviral + antitubercular agent	Nil	EBV, tubercle bacilli	Nil	1	1	0	Satisfactory
13	Antiviral + antibiotic	Dexamethasone	EBV	Nil	1	1	1	Fair
14	Acyclovir + antibiotic + antitubercular agent + low-dose hormone	Immunoglobulin	EBV	Nil	2	1	1	Satisfactory
15	Acyclovir + antibiotic + low-dose hormone	Immunoglobulin	EBV	Nil	3	2	1	Satisfactory

*EBV* Epstein–Barr virus, *mRS* modified Rankin scale

## Discussion

To gain insight into the clinical course of GFAP-A, we here reviewed the clinical characteristics, auxiliary examination results, treatment effects, and outcomes of 15 patients with GFAP-A. Our results suggest that autoimmune GFAP-A may be a spectrum disorder, with acute- or subacute-onset meningitis, encephalitis, and myelitis being the main phenotypes. In our patients with concomitant EBV infection, antiviral treatment was ineffective. As acute stage treatment, combined hormone and immunoglobulin therapy was superior to hormone or immunoglobulin pulse therapies alone.

GFAP is the major intermediate filament protein of mature astrocytes, and is involved in multiple functions, such as nerve regeneration, synaptic plasticity, and reactive gliosis [1]. Currently, there is still controversy about whether anti-GFAP is pathogenic and whether anti-GFAP antibodies are the responsible antibodies. Some reports indicate that anti-GFAP antibodies do not exert direct pathogenic effects by binding to antigens on cell surfaces in a manner similar to anti-AQP4‐IgG, which makes them a potential alternative marker for a cytotoxic T-cell-mediated autoimmune response [7, 8]. However, in an animal model study that used rat brain homogenate for subcutaneous injection, anti-GFAP antibodies were detected in serum, which was consistent with the pathological characteristics of biopsy and suggested the possible participation of GFAP-specific T cells in GFAP‐A onset. There are eight isomers of GFAP, and we suppose that some isomer-associated antibodies are pathogenic, while other isomers may be non-pathogenic [9]. Whether pathogenicity is associated with individualized genes in humans requires further investigation.

Autoimmune GFAP-A is characterized by a widely varying clinical presentations. For cases of acute encephalitis or meningoencephalitis type presenting with typical fever, headache, neck resistance, and impaired consciousness, we can accurately predict positive GFAP antibodies when combined with the characteristic symptoms of "tremor" and "bowel and Urinary dysfunction ". GFAP-A is a specific clinical syndrome. Given that male patients are often misdiagnosed with prostatic hyperplasia, tremor and urinary and bowel dysfunction can be regarded as the characteristic dual symptoms of autoimmune GFAP-A and may greatly aid in disease diagnosis. Tremor may be related to immune response-induced interference with dopamine uptake, storage, and release by astrocytes [10].

The CSF characteristics of GFAP-A are highly similar to those of tuberculous meningitis (TBM), which often leads to misdiagnosis and the unnecessary use of antitubercular treatment. Our study demonstrated that GFAP-A encephalitis possesses certain distinct characteristics. Based on our experience, we have summarized characteristics other than the common manifestations of encephalitis and meningitis, that can aid in distinguishing between GFAP-A and TBM, as shown in Table 7.

**Table 7:** The discrimination of GFAP-A and TBM

	GFAP-A	TBM
(1) Clinical presentations		
1. Postural tremor of differing severity in extremities	In all cases	Rare
2. Frequent micturition/urgent micturition/and (or) constipation of unknown cause during initial disease onset	In most cases	Rare
3. Rapid progression to quadriplegia	In certain cases	Rare
4. Ataxia in trunk or extremities	In most cases	Rare
(2) Inflammatory markers (initial disease onset)		
1. WBC	Almost normal	Mostly elevated
2. CRP	Almost normal	Significantly elevated
3. PCT	Mostly normal, or mildly elevated to not more than three times the upper limit of normal after multiple re-examinations	Mostly significantly elevated
4. ESR		Mostly elevated
(3) CSF		
Pressure	Mostly mild-to-moderate increases	Mostly moderate to severe increases
WBC and protein	Mild increase in WBC, usually mismatched with a significant increase in CSF protein	WBC and protein are significantly increased concurrently
EBV	More than half of patients may test positive	Concomitant infection is rare
(4) Auxiliary examinations		
EMG	Acute peripheral neuropathy may be present	Rare
EEG	Generalized abnormalities are found in more than half of cases	Mostly normal or mildly abnormal
Imaging	May be normal or indicative of the presence of multiple intracranial abnormalities; abnormalities may be observed in the splenium of the corpus callosum, certain patients may have abnormalities in the cervical spinal cord	Mostly enhancements in the meninges or cranial base
(5) Treatment effects		
Antitubercular agents	Ineffective	Effective
Immunoglobulin therapy	Effective or highly effective	Insignificant therapeutic effects
Hormone pulse therapy	Effective	Aggravates disease

*WBC* white blood cell, *CRP* C-reactive protein, *PCT* procalcitonin, *ESR* erythrocyte sedimentation rate, *EBV* Epstein–Barr virus, *TBM* tuberculous meningitis, *GFAP-A* glial fibrillary acidic protein astrocytopathy, *EMG* electromyography, *EEG* electroencephalography

A time lag was present in the presentation of anti-GFAP antibodies in some patients, which complicates disease diagnosis. Two patients exhibited antibody negativity in both serum and CSF at the first lumbar puncture at 7 days after disease onset, and two patients were misdiagnosed as having TBM, due to a low antibody titer during the initial onset, and received antitubercular therapy, which led to liver injury. The peak in antibody level also lagged behind symptomatic improvement after treatment. Therefore, future studies on a larger number of cases are needed to determine changes in antibody patterns. We also observed that eight patients who were clinically diagnosed with other diseases did not manifest any of the classic clinical presentations of autoimmune GFAP-A and exhibited anti-GFAP antibody positivity, which turned negative in subsequent re-examination. The causes of such phenomena are unknown, and their elucidation requires further follow-up observations.

In the present study, most patients were confirmed as having concomitant EBV infection in the CSF through mNGS and PCR (8/15), which provides further etiological evidence. However, antiviral therapy was proved to be ineffective. Therefore, patients with EBV infection should be highly suspected of having concomitant GFAP-A when the aforementioned typical nervous system symptoms are manifested. All suspected infectious diseases do not exclude the cases from our research for the reason that there is insufficient evidence to suggest an infectious disease. Some infections may be associated with the triggering of GFAP-A, but further confirmatory research is needed.

In some countries, reports have found concomitant tumors in 34% of cases [1, 3]. In the present study, no clear evidence of the presence of tumors was found in any patients during follow-up. Further follow-up will be performed to obtain data over a longer time period for confirmation.

The EMGs of four patients showed the concomitant presence of significant peripheral neuropathy with both motor and sensory nerve involvement and a decrease in the velocities and amplitudes of motor and sensory nerve conduction. Paul et al. [11] reported that serum anti-GFAP antibodies may participate in immune-mediated peripheral neuropathy. In the present study, the EMGs of these four patients suggested the presence of symmetric axonal injury in the four extremities, which was clinically manifested as acroparalysis and symptoms of numbness. Patient 1 also exhibited anti-sulfatide antibody positivity in serum, which was in agreement with the previous reports.

There is currently no clear guidance on the treatment regimens for autoimmune GFAP-A. Presently, the most widely used regimens during the acute stage are high-dose steroid hormone pulse therapy, intravenous γ-globulin injection, and plasma exchange. All patients showed steroid sensitivity. Some patients did have a lag in steroid treatment in the patients included in the study. The reason is that considering the side effects of high-dose steroids and the unclear diagnosis of some patients in the early stage, we need to wait for the investigation results to be confirmed and completely exclude tuberculous meningoencephalitis before starting high-dose steroid therapy. Therefore, we have several groups of treatment data, such as different doses of dexamethasone 5 mg and 10 mg, Immunoglobulin therapy alone proved, delayed steroid use, and sequential steroid treatment after globulin. We found that high-dose steroid therapy, such as methylprednisolone 500–1000 mg, was the effective protocol for patients with severe disease, which is consistent with the literature [7, 9]. For recurrent or refractory disease, additional use of immunosuppressants (e.g., mycophenolate mofetil or azathioprine), rituximab, or cyclophosphamide may be considered [7, 9, 12]. Among the patients of the present study, the first-choice treatment for those with concomitant EBV infection was acyclovir or ganciclovir alone, which proved to be ineffective. Therefore, when indications are lacking, immunoglobulin pulse therapy should be prioritized for early treatment. High-dose steroid initiate treatment should be administered to avoid further deterioration of the disease once the infectious disease was ruled out. None of the patients underwent plasma exchange or received immunosuppressant therapy. Based on the above observations, it can be deduced that combined hormone and immunoglobulin therapy was superior to hormone pulse therapy or immunoglobulin pulse therapy alone during the acute stage.

The overall long-term outcomes of the patients were satisfactory. Previous literature has reported recurrence in approximately 20–50% of patients [7]. In the present study, the longest follow-up duration was 2 years, but recurrence has not been observed in any of the patients to date.

## Conclusions

GFAP shares most of the prevalent features of encephalitis with its certain specificities, e.g., tremor bowel and urinary. Laboratory findings of the inflammatory index are not high in the early stages. Treatment is generally steroid-sensitive and it tends to have a better prognosis in the long term. Therefore, GFAP has certain characteristics of its own, and it may be a class of spectrum diseases.

In conclusion, the early discovery, diagnosis, and treatment of GFAP-A may lead to better outcomes, while delayed diagnosis may result in neurological impairment-related sequelae and poor outcomes. The summary of clinical characteristics presented in this study for the identification of GFAP-A had potential for widespread application, as it may aid in disease diagnosis and the establishment of diagnostic criteria.

## Data Availability

The datasets used, analyzed, or both during this study are available from the corresponding author upon reasonable request.
